# Cross-Presentation of Cell-Associated Antigens by MHC Class I in Dendritic Cell Subsets

**DOI:** 10.3389/fimmu.2015.00363

**Published:** 2015-07-17

**Authors:** Enric Gutiérrez-Martínez, Remi Planès, Giorgio Anselmi, Matthew Reynolds, Shinelle Menezes, Aimé Cézaire Adiko, Loredana Saveanu, Pierre Guermonprez

**Affiliations:** ^1^Laboratory of Phagocyte Immunobiology, Peter Gorer Department of Immunobiology, CMCBI, King’s College London, London, UK; ^2^Laboratory of Phagocyte Immunobiology, Peter Gorer Department of Immunobiology, Centre for Molecular & Cellular Biology of Inflammation (CMCBI), King’s College London, Paris, France; ^3^Sorbonne Paris Cité, Université Paris Diderot, Paris, France

**Keywords:** cross-presentation, cross-priming, dendritic cells, antigen processing, MHC class I, phagocytosis

## Abstract

Dendritic cells (DCs) have the unique ability to pick up dead cells carrying antigens in tissue and migrate to the lymph nodes where they can cross-present cell-associated antigens by MHC class I to CD8^+^ T cells. There is strong *in vivo* evidence that the mouse XCR1^+^ DCs subset acts as a key player in this process. The intracellular processes underlying cross-presentation remain controversial and several pathways have been proposed. Indeed, a wide number of studies have addressed the cellular process of cross-presentation *in vitro* using a variety of sources of antigen and antigen-presenting cells. Here, we review the *in vivo* and *in vitro* evidence supporting the current mechanistic models and disscuss their physiological relevance to the cross-presentation of cell-associated antigens by DCs subsets.

Cross-presentation is the process by which exogenous antigens captured by phagocytic antigen-presenting cells are processed and presented onto MHC-I molecules (1, 2). Early evidence supports the notion that cell-associated antigens are a physiological substrate for cross-presentation. Indeed, cross-presentation was discovered in the context of CD8^+^ T cell responses to grafts: Bevan et al. demonstrated that engraftment of H-2^b^ cells primes H-2^dxb^ F1 mice for a cytotoxic CD8^+^ T cell response against minor histocompatibility antigens restricted by H-2^d^ (1–3). Therefore, minor histocompatibility cellular antigens from the grafted cells are “cross-presented” by recipient antigen-presenting cells to CD8^+^ T cells, a process termed cross-priming because it is associated with productive CD8^+^ T cell activation (cf below). Later on, these findings were translated to tumor antigens as it was shown that MHC restriction elements were not required on the tumors to cross-prime tumor-specific CD8^+^ T cells (4, 5). Importantly, cross-priming antigen-presenting cells were found to originate in the bone marrow (4–6). The immunological outcome of cross-presentation depends on the DC activation state. Cross-priming is achieved by cross-presenting DCs that have received proper conditioning by pattern recognition receptor ligation or helper CD4^+^ T cells (7). By contrast, cross-presenting “steady-state” DCs mediate the functional inactivation of CD8^+^ T cells, a process termed cross-tolerization (8–10). Self-antigens like the one expressed in pancreatic β-cells, for example, were shown to enter the cross-presentation pathway constitutively for a tolerogenic outcome (8, 9). Therefore, cross-presentation might participate in the maintenance of peripheral tolerance to tissue-specific antigens (11). Also, cross-presentation of thymic epithelium self-antigens by bone marrow-derived cells can participate in the negative selection of auto-reactive CD8^+^ T cells (12). Importantly, cross-presentation might be mobilized to cross-prime CD8^+^ T cell responses specific to cell-associated viral antigens (4, 6, 13, 14). Heath and Carbone have proposed that the cross-presentation is an evolutionary response to evasion mechanisms associated with the direct infection of DCs by viruses (e.g., the inhibition of direct presentation or migratory behavior) (7). This view is supported by recent evidence that DCs can perform cross-presentation during infection without being infected (15). In all these situations (grafts, tumors, viral antigens), antigens are associated with cells reach the endocytic pathway of DCs where they gain access to the cross-presentation pathway for MHC-I presentation. *In vitro*, pioneer work from Bellone et al. (16) and Albert et al. (13) showed that the engulfment of dead cells by murine macrophages (16) or human monocyte-derived DCs (moDCs) (13) support the cross-presentation of cell-associated antigens by MHC-I. Quantitative studies demonstrated that association of protein antigen to dying cells considerably lowered the amount of antigen required for efficient cross-presentation *in vivo* (17). *In vivo*, cross-presentation is biased toward highly expressed cellular antigens and favored by cellular destruction (18), including cellular destruction dependent on cytotoxic CD8^+^ lymphocytes (18) or NK cells (19). Although other mechanisms might exist [nibbling, cytotoxicity by DCs (20–22)] these findings support the view that the phagocytosis of dead cells by DCs is an efficient mechanism underpinning the acquisition of antigens by cross-presenting APCs. Proteins, as opposed to proteasomal products (i.e., peptides), constitute the source of cell-associated antigens entering the cross-presentation pathway (23). Stable membrane proteins (24), in particular, are better cross-presented than soluble, short-lived cytosolic proteins or epitopes within signal peptides (25, 26). In summary, there is extensive *in vivo* evidence acquired in mouse models, and *in vitro* with human cells, supporting the relevance of cross-presentation by MHC-I for CD8^+^ T cell responses to cell-associated antigens. Here, we will review the current evidence identifying dendritic cells (DCs) as major players in the cross-presentation of cell-associated antigens and the mechanistic models that have been proposed to explain this phenomenon.

## Mouse and Human DC Subsets

Dendritic cells are classified as conventional DCs (cDCs) or plasmacytoid DCs (pDCs). cDCs represent a heterogeneous set of cells found in lymphoid and non-lymphoid tissues that: (i) pick-up and process antigens by MHC class I and class II molecules, (ii) activate naive CD4^+^ and CD8^+^ T cells (27–31), (iii) express a specific gene signature including the lineage-specific *Zbtb46* transcription factor (30, 32), (iv) rely on Flt3 receptor tyrosine kinase and its ligand for their development (33, 34), and (v) migrate toward T cell zones of lymphoid organs by using the chemokine receptor CCR7 (35, 36). In both mice and humans, cDCs can be classified into two subtypes, the XCR1^+^ DCs and the XCR1^−^ DCs (“cDC1” and “cDC2,” respectively, according to a recent nomenclature proposition)(37–39). In mice, the αE integrin CD103 is expressed on XCR1^+^ DCs with the notable exception of the gut where it is also expressed on a subset of XCR1^−^ DCs ontogenically distinct from cDC1 (40). Also, lymphoid organ-resident XCR1^+^ cDC1s express high levels of CD8α (40). cDC1s express some levels of the langerin protein also found in epidermal Langerhans cells (LCs) (41–43). Based on these findings, Langerin-DTR mice have been largely used as a model of DT-inducible conditional ablation of cDC1s (44–46). Overall, mouse cDC1s from various organs lymphoid or non-lymphoid share some common transcriptional programs and genetic requirements (e.g., Id2, IRF8, Batf3) (36, 40). In humans, XCR1^+^ cDC1s express BDCA3, while XCR1^−^ cDC2s express BDCA1/CD1c (37, 39, 47–49). Both murine and human cDC1s share a common transcriptional program characterized by high levels of TLR3, Clec9a/DNGR1 C-type lectin, and the IRF8 transcription factor (37, 39, 47–49). *In vivo* evidence obtained in *Batf3*^−/−^ mice (50) and *in vitro* silencing studies in human CD34^+^ progenitors identified Batf3 as a transcription factor relevant for cDC1 development in both species (51). Conversely, mouse and human cDC2s express high levels of IRF4 and TLR7 [mouse (52)] or TLR8 [humans (39, 53–56)]. Whereas IRF4 is required for the development of cDC2 in mice (57), it is not known if this holds true for human cDC2s. IRF4 is a master regulator of antigen presentation by major histocompatibility complex class II (MHC-II) through the induction of CIITA, the master transcription factor controlling the expression of MHC-II genes and accessory proteins (Ii, H-2DM) (58). Both cDC1 and cDC2 subsets are hematopoietic cells that develop from DC-committed, common DC precursors (CDPs) identified both in mice (59, 60) and more recently in humans (61). CDPs arise from common progenitors for DCs and monocytes (61, 62) and give rise to circulating precursors called pre-cDCS (63, 64). Finally, fate mapping studies (65, 66) and bar-coding of multipotent progenitors (67) identify cDCs as a *bona fide* hematopoietic lineage distinct from other mononuclear phagocytes and the lymphoid lineage.

Discrepancies between developmental abnormalities observed in cDC subsets in IRF8 mutant mice (57, 68, 69) and IRF8 mutant patients cast some doubt upon the actual level of orthology between human and mouse subsets. Indeed, *Irf8*^−/−^ mice are fully deficient in cDC1 (and pDCs and monocytes) but not cDC2. IRF8^R294C^ hypomorphic mutant mice (69) are also deficient in cDC1 (but not pDCs). By contrast, human genetic studies showed that patients with the IRF8^K108E^ mutation are deficient in both cDC1 and cDC2 (and monocytes), whereas a patient with the IRF8^T80E^ mutation selectively lacked circulating BDCA1^+^ cDC2 (70). Therefore, the conservation of developmental requirements between mouse cDC1 and human cDC1, if any, remains to be fully understood.

Plasmacytoid DCs represent a heterogeneous group of cells expressing specific markers in mice (PDCA-1, SiglecH) and humans (BDCA2/CD303, CD123). Mouse and human pDCs exhibit a high level of conservation in their gene expression pattern (71). Both rely on the Tcf4/E2-2 bHLH transcription factor (72) [and its Spi-B target gene (73, 74)] and can be generated from CDPs and other Flt3^+^ progenitors (61, 75–77).

Finally, moDCs represent a heterogeneous set of cells that are different from cDCs because: (i) they originate from monocytes and not pre-cDCs, (ii) they are absent at the steady state, (iii) *in vivo*, they rely on Ccr2-dependent egress of the bone marrow (78, 79). *In vivo*, moDCs arise in various inflammatory conditions triggered by exposure to foreign objects such as recall antigens (mBSA) (80), LPS (81), or during infections such as *Listeria monocytogenes* (82, 83). *Listeria*-induced iNOS^+^ moDCs are independent of GM-CSF (83) while mBSA-dependent moDCs rely on GM-CSF (80). *In vitro* generated GM-CSF-derived DCs (84) are a popular source of DCs for cellular studies even if they are developmentally distinct from cDCs (85).

## *In vivo* Evidence for the Role of Murine cDC1 in Cross-Presentation

If cross-presentation can be obtained using multiple antigen-presenting cells *in vitro*, including moDCs or macrophages for example, the available *in vivo* evidence suggests that cross-presentation is mostly performed by the mouse CD8^+^/CD103^+^ subset of cDCs (cDC1s). Evidence supporting this paradigm was obtained by analyzing *ex vivo* MHC-I peptide complexes on spleen DCs sorted from mice that had previously received an intravenous injection of OVA antigen-loaded cells (86). CD8α^+^CD11b^−^ cDC1 but not the CD8α^−^CD11b^+^ cDC2 were found to perform cross-presentation. cDC1s were also involved in the constitutive cross-presentation of a pancreatic model antigen (RIP-OVA) (8, 9, 87). Also, lung cDC1s pick up intranasally delivered soluble antigens or cell-associated antigens, transport them to mediastinal lymph nodes, and perform cross-presentation (88, 89).

Which DC subsets perform cross-presentation during viral infections? Allan et al. have shown that upon HSV skin infection, skin migratory DCs, but not radio-resistant LCs, were responsible for the early transport of antigens to the lymph node, where antigen is transferred to lymphoid resident CD8^+^ cDC1s, mediating CD8^+^ T cell priming (90, 91). Similar findings advocating a major role for lymphoid organ-resident cDC1s cross-presenting cell-associated antigens have been reported in multiple infection systems (92). Later on, this idea was challenged by Bedoui et al. who addressed the efficiency of *ex vivo* presentation by the different DC subsets after 5 days of infection with HSV-1 (93). The isolation of DCs from axillary lymph nodes draining a site of secondary infection (associated with viral recrudescence) demonstrated that the CD103^+^ migratory cDC1s had a predominant role in the presentation of viral antigens to specific CD8^+^ T cells *ex vivo* (93). However, *ex vivo* T cell activation in this system does not discriminate between cross-presentation and direct presentation. Bedoui et al. obtained formal evidence of the role of migratory cDC1 in cross-presentation using a membrane-associated form of ovalbumin (OVA) expressed in epidermal keratinocytes (93). Finally, selective depletion of migrating langerin^+^ cDC1s (but not LCs) was shown to be required for the constitutive cross-presentation of keratinocyte antigens formally demonstrating the key role of migratory cDC1s in cross-presentation of cell-associated antigens from the periphery (46).

Upon lung Influenza virus infection, there is an increase in the population of langerin^+^CD103^+^CD11b^−^CD11c^+^ cDC1s in the mediastinal lymph node (94). The depletion of this population of cells in Langerin-DTR mice leads to a decrease in the clearance of Influenza virus together with a significant decrease in CD8^+^ cytotoxic responses (94). Importantly, a subsequent study showed that among all the DC subsets in mice infected with a recombinant influenza virus, only the lung CD103^+^ cDC1s carried antigens from a recombinant Influenza virus expressing a green fluorescent protein (GFP) fused to NS1, a non-structural protein of the virus, to the draining lymph node (15). Importantly, CD103^+^ cDC1s carrying GFP^+^ material derived from infected cells were not infected. Therefore, this study elegantly demonstrated that cross-priming DCs mediate CCR7-dependent delivery of cell-associated viral antigen to draining lymph nodes without being infected (15). Also, depletion of cDC1s in *Batf3*^−/−^ mice abrogates CD8^+^ T cell responses to West Nile virus, but the contribution of cross-presentation in this process is not formally established (50). Altogether, the migratory CD103^+^ cDC1s have emerged as a pivotal DC population in the control of CD8^+^ T cell responses, especially in the context of viral infections.

CD103^+^ DCs might also have a role in other types of infections. Splenic CD8^+^ cDC1s isolated from the spleens of mice infected with *Plasmodium berghei* ANKA were much more efficient in cross-presenting malaria antigens than cDC2s (95). As a consequence, depletion of cDC1s in Langerin-DTR mice (96) or *Clec9a*-DTR (97) mice abrogates immuno-pathological CD8^+^ T cell responses associated with *P. berghei* ANKA infection. In the case of malaria infection, cross-presentation is absolutely required for CD8^+^ T cell responses, since parasites invade only red blood cells devoid of MHC class I(98).

### Are other DCs types involved in cross-presentation *In vivo*?

Monocyte-derived DCs generated in inflammatory conditions *in vivo* are capable of cross-presenting, to some extent, soluble OVA or OVA expressed by *E.coli* (81, 99). However, it is unclear if this mode of cross-presentation is relevant for the induction of CD8^+^ T cells *in vivo*, in particular against cell-associated antigens. Immunization based on skin inflammation was shown to involve CCR6-dependent inflammatory cells that cross-present to CD8^+^ T cells (100). Also, moDCs might support the expansion of antigen-experienced CD8^+^ T cells in the periphery (101). If these studies (100, 101) implicate moDCs in antigen presentation to CD8^+^ T cells, it remains unknown if moDCs can perform the cross-presentation of cell-associated antigens *in vivo*. Interestingly, it has been shown that circulating CD14 monocytes of chronic HBV patients contain big amounts of viral antigen that are mobilized to elicit T cell proliferation when they are differentiated into moDCs (102).

In spite of the lack of cross-presenting activity in steady-state conditions, one study showed that upon stimulation with TLR7 agonists *in vivo* or *in vitro*, the capacity of splenic pDCs to cross-present antigens was increased suggesting that they might contribute to cross-presentation *in vivo* (103). However, depletion of pDCs by the injection of 120G8 antibodies does not affect CD8^+^ CTL responses to Influenza (94). Likewise, the administration of OVA coupled to a PDCA-1 antibody which would direct the antigens to the pDCs does not elicit CD8^+^ responses in the absence of cDCs (104). Taken together, there is little evidence for a role of pDCs in cross-presentation *in vivo* despite the recent evidence of their contribution to some CD8^+^ anti-viral responses, through the orchestration of innate immune responses (105, 106).

## Are Human cDC1 Specialized in Cross-Presentation?

There is currently some controversy on whether human cDC1s are endowed with a better cross-presenting ability than cDC2s. Many reports have analyzed the cross-presentation of antigens associated with necrotic cells to antigen-specific CD8^+^ T cell clones *in vitro*. Using HIV-infected necrotic cells, Crozat et al. reported that cDC1s are more efficient at stimulating a CD8^+^ T cell line specific for the HIV protein pol (37). Jongbloed et al. analyzed cross-presentation of HCMV-infected necrotic fibroblast to pp65-specific CD8^+^ T cells and reported similar findings using DCs purified from blood (48). Finally, Bachem et al. also concluded the superiority of blood cDC1s in cross-presenting cell-associated antigens from freeze-thawed transfectants expressing the pp65 HCMV protein (47). By analyzing the tonsil-derived cDC1s, cDC2s, and pDCs, Segura et al. concluded that both cDC1s and cDC2s have a similar ability to cross-present cell-associated antigens that is not shared by pDCs (107, 108). In contrast, Tel et al. showed that pDCs can cross-present receptor-targeted Ags to cytotoxic T lymphocytes so efficiently as cDC1 (109).

Finally, Haniffa et al. have identified a skin CD141^+^ cDC1 subset capable of cross-presenting a hepatitis B surface antigen (HBsAg) to specific CD8^+^ T cell clones upon stimulation with TLR3 agonists and exposure to a cytokine maturation cocktail (110).

Some experiments, however, support the idea that cDC1s might be endowed with a better ability to cross-present exogenous antigens independently of dead cell engulfment. Indeed, two studies have reported that cDC1s have a better ability to cross-present soluble proteins to antigen-specific CD8^+^ T cell clones (47, 48). This result is in stark contrast to the data of Segura et al. who did not identify a detectable cross-presentation activity for a long peptide in circulating, non-activated cDC1s and cDC2s (107). Both cDC1s and cDC2s from the tonsil, however, were found to be able to cross-present soluble antigens (long peptides and protein antigens) efficiently, together with pDCs (108). Also, pDCs have been reported to cross-present vaccinal lipopeptides and HIV-1 antigens from apoptotic cells to specific CD8^+^ T lymphocytes as efficiently as cDC2 (111).

Dendritic cells derived from CD34^+^ hematopoietic progenitors are increasingly used as a convenient model for the study of cross-presentation by DCs. Pioneering work by Poulin et al. has characterized the cDC1 progeny of CD34^+^ progenitors differentiated in a Flt3L/SCF/GMCSF/IL4 cytokine cocktail (49). Poulin et al. have shown that cDC1-like TLR3^+^Clec9a^+^ cells were capable of cross-presenting Melan-A long peptide upon polyI:C activation (49). Using a similar culture system, by systematic comparison of cDC1s and cDC2s, Balan et al. have found that XCR1^+^TLR3^+^Clec9a^+^ cDC1s have a better ability to cross-present cell-associated HCMV pp65 associated with K562 cells than BDCA1/CD1c^+^ cDC2s (112). These data contrast with the study of Proietto et al. showed that both cDC1 and cDC2 obtained in Flt3L/TPO cocktail do not display differences in the cross-presentation capability of an Influenza virus protein to a specific CD8^+^ T cell (113). Finally, Segura and Kletchevksy have firmly established the cross-presentation abilities of CD1a^+^ DCs derived from CD34^+^ cord blood progenitors cultivated in GMCSF/Flt3L/TNF-α (108, 114). The developmental and phenotypic relationship of these cells that share a lot of features with epidermal LCs (114) raise some interesting questions on the regulation of cross-presentation *in vivo* in the human system. Supporting this idea, another group showed that human LCs were, at steady state, more efficient than dermal DCs in cross-presenting long peptides to CD8^+^ T cells, (115).

## Is the Ability to Engulf Dead Cells Associated with a Specific DC Subset?

Den Haan et al. first identified the mouse DC1 subset as the main phagocytic subset involved in the phagocytic uptake of dead cells *in situ* (86). Iyoda et al. later extended these findings in the context of NK-mediated killing of allogeneic targets (19). More recently, Desch et al. have identified that mouse lung cDC1s have a specific ability to pick up intranasally delivered dead cells (89).

In summary, there is a general agreement on the fact that murine cDC1s have a higher ability to pick up dead cells as compared to cDC2s. In humans, however, the situation is not that simple. Jongbloed et al. found that the superiority of DC1s (as compared to cDC2s) has not been attributed to a specific ability to pick up material from dead cells (48). By contrast, Segura et al. found that both cDC1s and cDC2s (but not pDCs) were found capable of engulfing dead cell material from fluorescently necrotic cells (107, 108). Despite their ability to perform cross-presentation of long peptides and proteins with the same efficiency as cDC1s or cDC2s, pDCs are poorly able to engulf material from dead cells and to cross-present cell-associated antigens (107, 108). In summary, unlike in the mouse system, both human cDC1 and cDC2 seem to be able to pick up necrotic dead cell material with no obvious differences in efficiency.

### What are the receptors implicated in the uptake of dead cells by cDCs?

The mechanisms of dead cell engulfment by macrophages are complex and involve multiple receptors that have cooperative or redundant roles (116). Phosphatidylserine exposure associated with cell death engages directly macrophage receptors (e.g., TIM4, CD36) and also leads to the deposition of multiple opsonins (e.g., MFGE8, Gas6, protein S) engaging various macrophage receptors (e.g., alphaV integrins or MerTK/Axl tyrosine kinases). Dead cell uptake in macrophages requires a coordinated process of direct recognition of “eat-me” signals and the subsequent activation of intracellular signaling pathways. For example, TIM4 receptors mediate the uptake of dead cells to the macrophage plasma membrane by direct recognition of phosphatidylserine (117), whereas tyrosine kinases like MerTK triggers a signaling cascade that leads to an actin-remodeling process promoting engulfment (118). Initial work by Albert et al. showed that the alphaVbeta5 (αVβ5) integrin and CD36 scavenger receptor play an important role in dead cell uptake by human moDCs (119). However, murine DC1 spleen cells from *Cd36*^−/−^ and β*3*β*5*^−/−^ mice are not deficient in the uptake of dead cells and cross-presentation (120, 121). By contrast, neutralizing antibodies to αV integrins and CD36 inhibits efferocytosis by human splenic CD141^+^ cDC1s (122). Therefore, the involvement of CD36 and aV integrins in dead cell uptake by DCs remains controversial, as it is not clear if DCs and macrophages share similar mechanisms to perform efferocytosis. In this sense, evidence for specific cell-type efferocytic mechanisms are reinforced by a study showing that the inhibition of phosphatidylserine recognition *in vivo* impairs macrophage dead cell uptake, but promotes efferocytosis by spleen CD8α^+^ DCs (123).

Mouse cDC1s express DEC205 (124) and DNGR1/Clec9a (125) lectins and both have the ability to bind to dead cells. Analysis of *DEC205*^−/−^ (19) failed to reveal any default in the internalization of dead cells by cDC1s. Clec9a/DNGR1, in particular, has been shown to bind filamentous actin (126–128) and is also selectively expressed by mouse and human cDC1s (49, 51, 129, 130). Surprisingly, in spite of its ability to bind to necrotic cells by engaging filamentous actin (126–128), Clec9a/DNGR1 is not controlling the uptake of dead cells by the cDC1s DCs (125, 129, 130).

More recently, TIM3 has been involved in the phagocytosis of apoptotic cells by the mouse DCs (131). Anti-TIM3 antibodies inhibit efferocytosis by mouse cDC1s *in vitro* and *in vivo*, and partially decrease cross-presentation as well (122, 131). Although the role of TIM3 in cross-presentation by human cDCs has not been assessed, a recent paper has shown that TIM3 is highly expressed in tumor-associated cDCs in humans (132). Another scavenger receptor, SCARF-1, has also been involved in efferocytosis in mouse cDC1 (133) although the administration of neutralizing anti-SCARF-1 antibodies does not have any effect on the efferocytosis by human spleen cDC1 (122). Whether SCARF-1 plays a role in cross-presentation has not been established. Finally, the Axl tyrosine kinase has also been involved in efferocytosis by DCs (122). Axl would mediate the recognition of opsonized dead cells while RANBP9 mediates its interaction with the LRP1 phagocytic receptor (122). Inactivation of either member of this complex leads to pronounced reduction in dead cell uptake by mouse cDC1 with the subsequent decrease in cross-presentation of cell-associated antigens. Importantly, antibody blockade indicates that Axl and Lrp1 are also needed for the uptake of dead cells by human splenic cDC1 (122).

In summary, mouse and human cDC subsets might recruit overlapping as well as distinct set of receptors to mediate the engulfment of dead cells. Whether the nature of cell death might influence these choices and the cellular consequences for cross-presentation of cell-associated antigens remains to be analyzed.

## Does Antigen Targeting to Endocytic Receptors Trigger Cross-Presentation in all Subsets?

If murine cDC1s have a clear advantage in dead cell uptake as compared to cDC2s, it is conceivable that additional intracellular processes might also contribute to efficient cross-presentation of cell-associated antigens. In support of this notion, Schnorrer et al. have shown that phagocytic cDC1 cross-presents OVA more efficiently than cDC2, even at normalized amounts of phagocytozed antigen (134). Is it possible to overcome these subset-specific differences in cross-presentation efficiency by increasing the efficiency of antigen loading by targeting endocytic receptors? For example, experiments from the Cresswell laboratory have demonstrated that 293T cells rendered phagocytic by enforced expression of FcgRIIA are capable of cross-presenting antibody-targeted antigens (135). Therefore, it is tempting to conclude that every cell is able to perform cross-presentation provided that exogenous antigens are delivered efficiently inside the endocytic pathway. Complex particles like bacteria (136) or yeast (137), engaging multiple receptors and triggering cell activation, overcome the superiority of mouse cDC1 to perform cross-presentation, at least *in vitro* (138). However, by carefully measuring the rates of antigen uptake, Kamphorst et al. were able to demonstrate the superiority of cross presentation efficiency by cDCs (cDC1 or cDC2) over GMCSF-induced moDCs regardless of the route of antigen capture (receptor-mediated endocytosis, macro-pinocytosis or phagocytosis) (139). Therefore, cDCs appear to be endowed with a kind of capability to perform cross-presentation, irrespectively of cDC1/cDC2 subset, more efficiently than other phagocytic cell types even if antigen is delivered through receptor-mediated endocytosis (139).

Elegant experiments from the Nussenzweig laboratory have established that targeting of exogenous antigen to mouse DEC205 via antigen-antibody conjugates leads to efficient antigen cross-presentation in cDC1 cells that selectively express DEC205 (10, 140). When targeted to cDC2s, the same antigen is not cross-presented efficiently (140). Transgenic expression of human DEC205 in both cDC1 and cDC2 cells using the CD11c promoter and antibody-mediated targeting was instrumental in showing that (i) efficient capture of OVA by huDEC205 leads to efficient cross-presentation by both cDC1 and cDC2 and (ii) in the same cells (cDC2), huDEC205 leads to better cross-presentation than DCIR2 targeting (139). Unlike mouse DEC205, human DEC205 is expressed in both human cDC1s and cDC2s. Efficient cross-presentation is achieved in both cDC1s and cDC2s when antigen is targeted to CD40 or CD11c but not DEC205 despite the ability of DEC205 to trigger efficient internalization of antibody-antigen conjugates in both subsets (141, 142). In conclusion, the level of subset specificity achieved by receptor targeting in cross-presentation is fully dependent on the endocytic receptor used (139, 141, 142). Overall, cDC2 are largely able to perform efficient cross-presentation upon targeting of antigen to some specific receptors (139, 141, 142). These findings are important for the design of vaccines (143), but one may wonder if receptor-dependent cross-presentation by cDC2s is physiologically relevant. In physiological settings, antibody-mediated uptake by FcγR might significantly boost the cross-presentation of antigens by cDC2s. This was shown *in vitro*, for example, with the cDC2-like D1 cell line or moDCs (144) and *in vivo* after the injection of immune complexes (145). This mode of uptake might serve the cross-presentation of cell-associated antigens when antibodies raised against cell surface antigens mediate cellular engulfment (146). This situation might be important in the context of immune responses to viral infections when antibodies might bind viral glycoproteins at the surface of infected cells and promote the cross-presentation of cell-associated viral antigens. Also, antibodies against model membrane antigens expressed in pancreatic β cells activate its cross-presentation *in vivo* (147).

## Cytosolic Versus Vacuolar Cross-Presentation

What are the intracellular mechanisms of antigen processing and presentation underpinning cross-presentation? Currently, there are two main models explaining how antigens can be processed to load MHC-I receptors for cross-presentation. These models are referred to as “cytosolic” and “vacuolar” pathways. In the cytosolic model, antigens are released from the lumen of phagocytic compartments to the cytoplasm where they are further processed into short peptides by the proteasome (148). The transporters associated with antigen processing (TAP1/TAP2) translocate proteasomal peptide products to the lumen of the MHC class I loading compartment where they gain access to the MHC-I loading machinery including tapasin, ERp57, and calreticulin (148). The first evidence supporting the so-called cytosolic model came from studies using antigen associated with beads and macrophages as the source of antigen-presenting cells (149). Importantly, this study demonstrates the existence of a phagosome-to-cytosol transport pathway. Indeed, coupling of the gelsolin protein toxin to beads was sufficient to mediate its cytosolic delivery probed functionally by translational inhibition (149). Cross-presentation of phagocytosed antigen was inhibited by proteasome inhibitors, genetic inactivation of the TAP1/TAP2 transporters and Golgi disruption by brefeldin A, a Golgi-disrupting agent (149). These experiments constitute the foundation of the cytosolic model of cross-presentation in which phagocytosed antigens are processed within the cytosol by the proteasome in a similar way to endogenous antigens. TAP requirement for cross-presentation has also been identified for cell-associated antigens *in vivo* (6, 150). Favereau et al. have shown that the cross-presentation of cell-associated antigens by human moDCs uses the cytosolic pathway (151). However, the criterion of TAP requirement has to be considered with some caution since genetic inactivation of TAP1 or TAP2 destabillizes MHC-I and impairs its transport at the cell surface (152). As a consequence, low-temperature incubations, which restore the levels of MHC-I at the plasma membrane in TAP^−/−^ cells, can rescue the cross-presentation of phagocytosed antigens (153, 154). In addition to TAP requirement, experimental evidence of the cytosolic pathway relies on pharmacological proteasome inhibition.

In the vacuolar pathway, extracellular antigens are internalized and degraded in the endosomal compartments by lysosomal enzymes, and the resulting peptides are loaded onto MHC-I molecules within the endosomal compartment by a process akin to MHC-II presentation. Pioneering work from the Harding laboratory showed that cross-presentation can be achieved in macrophages in the absence of functional TAP transporters and in a way that is insensitive to pharmacological inhibition of the proteasome (155). Later on, TAP-independent cross-presentation was shown to involve cathepsin S (156). It is generally admitted that the vacuolar form of cross-presentation is associated with high levels of antigen delivered in the endocytic pathway. Whether this might be a physiological way to handle cellular antigens, or perhaps the most abundant of them, remains to be established.

## Antigen Retention in the Endocytic Pathway of Cross-Presenting cDCs

There is a wealth of evidence supporting the notion that limited endo-lysosomal proteolysis of engulfed antigens promotes their efficient cross-presentation by cDCs. Overall, limited endo-lysosomal proteolysis is associated with the DC lineage (157, 158). An elegant study by Delamarre et al. showed that GM-CSF DCs, splenic DCs and DCs from lymphoid organs expressed lower levels of lysosomal proteases than *in vitro* differentiated macrophages, splenic, lymphoid, and peritoneal macrophages. This correlates with a decrease in the capacity of DCs to degrade soluble proteins *in vitro* and *in vivo* (158). These findings were extended to human DCs derived from CD34^+^ hematopoietic progenitor cells or isolated from peripheral blood, which, in comparison with macrophages, are also protease poor (159). Overall, human cDCs express less cathepsin B, L, and S than moDCs, and both murine and human cDC2s express slightly more proteases than cDC1s (140, 142).

In addition to their low content in proteases, DCs also limit the activity of acid-dependent lysosomal proteases by maintaining a relatively alkaline endocytic pathway. Pharmacological inhibition of endocytic acidification by treatment with cloroquine, ammonium chloride, or protease inhibitors boosts cross-presentation of endocytosed proteins in human moDCs or cDC2 (141, 160, 161). The relatively high endo/phagosomal pH of DCs is mainly explained by two factors.

First, in immature DCs an important fraction of the cytosolic subunits of the vacuolar ATPase (V1 sector) appears dissociated from the trans-membrane V0 sector. As a proper assembly of both subunits is needed for the function of the V-ATPase, the higher pH can be partially explained by a deficient transport of protons to the lumen of the lysosome (162). Activation of DC maturation *in vitro* promotes the assembly of the complex and its role in luminal acidification (162). Interestingly, the phosphatidylinositol 3-kinase/mTOR pathway has recently been identified as an upstream regulator of the V-ATPase assembly during DC activation (163).

Second, the NADPH oxidase regulates phagosomal acidification in murine and human GM-CSF moDCs and CD8α^+^ cDC1s (164–166). The NOX2 complex is recruited to the phagosomal membrane and produces reactive oxygen species (ROS) that activates proton consumption and induces lumenal alkalinization (164, 166). As a consequence, both mice and human DCs deficient in functional NADPH oxidase have reduced cross-presentation (164–166). Recruitment of the Rac2 GTPase to phagosomes would support cDC1 NADPH oxidase at phagosomal membranes (166). The relevance of the Rac2/NAPDH oxidase complex on the cross-presentation of cell-associated antigens *in vivo* remains to be tested.

Another level of regulation of the phagocytic pathway involves the endoplasmic reticulum (ER)-resident 47 kDa interferon-inducible GTPase, Irgm3, and its Perlipin2/Adrp partner (167). Irgm3 is expressed in both mouse cDC1 and cDC2s subsets and further induced upon TLR3 activation (167). Irgm3 associates with the ER and lipid bodies (LBs) of DCs where it directly interacts with the LB coat protein Perilipin2 (167). The depletion of Irgm3 or Perilipin2 decreases the number of LBs at steady state and abrogates their induction upon IFN_ɣ_ and poly:IC treatments, whereas Irgm3 over-expression does the contrary. Importantly, the splenic cDC1s show higher steady-state levels of LBs than the cDC2s (167), which can be explained by higher Perilipin2 levels (53, 140, 167). The reduction of LBs by pharmacological treatments or genetic inactivation of Perilipin2 or Irgm3 inhibits cross-presentation by mouse cDC1, including the cross-presentation of cell-associated antigens *in vivo* (167). Interestingly, *Irgm3*^−/−^ moDCs have an accelerated phagolysosomal maturation as compared to their WT counterparts. The mechanisms underlying this effect are unknown, but a direct association between LB and the phagosomal membranes has been demonstrated in moDCs (167) and other cells (168). Interestingly, the NOX2 complex protein gp91^phox^ controls the recruitment of the cytosolic phospholipase A2 (PLA2) and LBs themselves to phagosomes (169, 170). An interesting hypothesis is that LBs might provide a lipid source for the formation of the NOX2 activator arachidonic acid (AA) in a PLA2-dependent manner (171, 172). Therefore, it is conceivable that NOX2 and LBs might cooperate to regulate oxidative metabolism with consequences for antigen proteolysis and, *in fine*, cross-presentation.

Finally, “antigen retention” early endocytic compartments segregated from the normal progression of endosomes to lysosomes have been described in DC lines after macro-pinocytosis or antibody-mediated uptake of soluble antigens (173, 174). Antigen retention compartments might provide a long-term source of antigen for the continuous loading of MHC-I receptors (173, 174). Burgdorf and Kurts found that mannose receptor targeting, but not fluid phase uptake, leads to prolonged antigen accumulation of antigen in antigen retention compartments with slow rates of maturation to lysosomes (175–177). This compartment is accessible to TAP and MHC class I loading complex upon TLR stimulation (176) (see below). Moreover, they can acquire the MHC class I antigen trimming enzyme insulin responsive aminopeptidase (IRAP), since mannose receptor endosomes partially overlap with the vesicular compartment described by IRAP, an enzyme that interacts with MHC class I molecules and performs antigen trimming in endosomes, in a model of proteasome-dependent cross-presentation (178). In the absence of IRAP, *in vivo* cross-presentation of antigens targeted to mannose receptors is compromised, underlining the functional relevance of mannose receptor and IRAP endosomes overlapping (178). However, there is a controversy regarding the relevance of this pathway for the uptake of OVA by spleen cDCs, specifically cDC1 (99, 177, 179). More generally, one might wonder if mannose receptor-targeted IRAP^+^ endosomes are of relevance regarding the cross-presentation and routing of cell-associated antigens during the engulfment of dead cells, which might be processed in the phagosome. In the phagosome, MHC class I ligand production is usually the result of a cooperative action of cytosolic proteasome, ER-resident trimming aminopeptidases and IRAP (180).

Dendritic cells receptors involved in dead cell uptake or sensing might regulate phagosome maturation to promote antigen retention for cross-presentation. In support of this notion, Sancho et al. have shown that *Clec9a*^−/−^ cDC1s have reduced cross-presentation of cell-associated antigen, *in vitro* and *in vivo* (125). By contrast, *Clec9a*^−/−^ cDC1s normally cross-present antigen coupled to latex beads suggesting a cargo-specific process (125, 181). Ligation of danger-associated molecular patterns found in dead cells like filamentous actin might act as a regulator of the trafficking of dead cells in cDC1s (126, 182). Supporting this notion, DNGR1/Clec9a deficiency inhibits the accumulation of Rab5a and Rab11 markers to the dead cell phagosome (182). A quantitative analysis of dead cell-containing phagosomes has been performed by the Janssen laboratory (183, 184). These authors have shown that cell remnants captured by murine splenic cDC1s, in particular a CD8α^−^Clec9a^+^ subset of cDC1s, have a smaller size and a longer persistence (up to 20 h) as compared to the ones captured by spleen cDC2 (183, 184). Interestingly, endocytic compartments carrying dead cell debris remains poorly acidified and retain early endocytic markers such EEA1 (183, 184). These *in vitro* data are consistent with the fact that cell debris carrying intact GFP from infected cells engulfed in the lung could still be found in non-infected, phagocytic cDC1 having reached lung draining lymph nodes (15). Overall, antigen retention by migratory cDCs might enable the coordination of antigen presentation/cross-presentation with migration from antigen sampling sites (tissues) to the T cell areas of the lymph nodes. However, the nature of antigen retention compartments and the mechanisms regulating the access of cell-associated antigens to this compartment remains to be addressed in more detail.

## The Translocation of Phagosomal Antigens Inside the Cytosol in cDCs Subsets

Initial work from the Watts laboratory identified growth factor-induced macro-pinocytosis as an efficient, actin-dependent, endocytic process leading to the export of captured antigens to the cytosol (185). Importantly, immature moDCs and cDCs were found to display constitutively active macro-pinocytosis mediating the delivery of exogenous antigens to the cytosol for cross-presentation (186, 187). Later on, morphological evidence of cytosolic access of protein antigens targeted to Fc-receptors and soluble dextrans were reported in DCs (188, 189). Importantly, antigen transport to the cytosol was found to be specific for DCs since it was not observed in macrophages (188).

Is the cytosolic pathway for cross-presentation active in specific cDCs subsets *in vivo*? Plamowski et al. have shown *in vivo* that cross-priming against an HY-encoded minor histocompatibility antigen is reduced in mice deficient for some immune-proteasome subunits (LMP7^−/−^) (190). This study represents a unique *in vivo*, indirect genetic demonstration of the involvement of the cytosolic pathway in the cross-priming of CD8^+^ T cells toward cell-associated antigens. The most compelling *in vivo* direct evidence of cytosolic export comes from a work in which mice were injected with soluble cytochrome *c* protein. Following this treatment, Apaf-1-dependent apoptosis was used as a readout of cytochrome *c* release to the cytosol (191). Lin et al. observed a selective depletion of spleen cDC1s (CD8^+^CD103^+^) upon cytochrome *c* depletion. These experiments suggest that cDC1s might be endowed with a specific ability to release antigen from the endocytic pathway inside the cytosol. Do these findings translate to humans cDC1s? This is not completely clear. Indeed, cross-presentation by human cDC1s is largely blocked by inhibitors of the proteasome supporting the use of soluble antigens in the cytosolic pathway (48, 108). Segura et al. have used an elegant β-lactamase-based fluorescent assay to monitor the delivery of soluble proteins to the cytosol of DCs (108). Using this assay they found that cDC1, cDC2, and pDCs (but not CD14^+^ macrophages) all perform the cytosolic export of endocytosed antigens efficiently (108). This elegant assay might selectively visualize early events of translocation, before possible destabilization of the enzyme by endo-lysosomal proteolysis. Distinct conclusions were obtained by Cohn et al. who report that cDC1s, but not cDC2s, do cross-present antigens when they access late endocytic compartments (142). This was exemplified with fusion-defective viruses, *Listeria*-encoded antigens from translocation-defective (LLO^−^) strains or DEC205 receptor targeting (142). An interpretation of these important findings is that cytosolic cross-presentation could be mediated by two pathways: an early endocytic cross-presentation pathway which is functional in both cDC1 and cDC2s, and a late endocytic pathway more specifically associated with cDC1s (141, 142). The later process could imply the existence of specific lysosomal mechanisms for efficient antigen export only present in the cDC1. Alternatively, lysosomal processing of antigens could occur more slowly in the cDC1 allowing their cytosolic export before complete lumenal degradation. Further mechanistic studies are needed to decipher whether the translocation of antigens to the cytosol is an active process associated with the endocytic membranes of cDCs or a passive process associated with the accumulation of non-degraded proteins in endocytic compartments. In support of the latter, experimental inhibition of protein degradation leads to improved transport in the cytosol (160, 189).

## A Role for Endoplasmic Reticulum-Associated Degradation in the Translocation of Phagocytic Antigens into the Cytosol by cDCs

With the existence of a phagosome-to-cytosol pathway being well accepted, the mechanisms underlying the translocation of antigens across the endocytic membrane are the focus of intense investigations. In multiple experimental systems, enzymatically active proteins have been found in cytosolic extracts, suggesting that the cytosolic translocation might not necessarily involve protein unfolding. The disruption of endocytic membranes might be put forward as a hypothesis. Indeed, experimental osmotic disruption of endo-lysosomes is sufficient to trigger cross-presentation of exogenous antigens (192). However, disruption of endo-lysosomal membranes is associated with the induction of cell death by lysosomal proteases [e.g., cathepsin (193)]. A popular hypothesis explaining the transport of antigens to the cytosol involves the access of endocytic antigens to translocation processes associated with the ER. The active transport of ER-associated ill-folded proteins inside the cytosol for proteasomal degradation (ERAD) in particular has been speculated to regulate cross-presentation by MHC-I. The channel protein sec61, which has been involved in the export of mis-folded proteins to the cytosol as part of the ERAD machinery (194, 195) is present in the phagosomal membrane together with other ER markers (196–198). More recent studies have shown that the ER-resident SNARE sec22b is directly involved in the recruitment of ER membranes to the phagosome, possibly favoring the onset of ERAD of luminal antigens (199). The specific involvement of sec61 in the translocation of peptides to the cytosol has been demonstrated *in vitro* using assays in which the export of radio-labeled peptides from microsomes was inhibited in the presence of the sec61 inhibitor *Pseudomonas aeruginosa* exotoxin A (ExoA) (200). The knockdown of sec61 in the DC2.4 moDCs line decreased cross-presentation of soluble OVA supporting that it is a role for sec61 in cross-presentation (201). A more recent paper has demonstrated that sec61 is recruited to endosomes carrying OVA antigen and that such recruitment depends on TRIF signaling. Inhibition of sec61 release from the ER to the endosomes using a specific antibody against sec61 fused to an ER retention sequence impairs cross-presentation and antigen export to the cytosol in DC2.4 and BMDCs (202).

However, the involvement of sec61 in cross-presentation by the human DC subsets is still controversial, as it has been reported that depletion of sec61 or the use of ExoA did not have any impact in cross-presentation of an HLA-A2-restricted epitope by human DC differentiated from monocytes (203). Another member of the ERAD machinery related to cytosolic export of antigens during cross-presentation is the soluble AAA ATPase p97, which is thought to provide the required energy for the passage of proteins through ER membranes (204). The addition of purified recombinant p97, but not a dominant negative form of the protein, is enough to promote the export of proteins from purified luciferase-loaded phagosomes even in the absence of other cytosolic factors (205). In functional terms, cells expressing a dominant negative form of p97 show a decrease in cross-presentation of soluble OVA (205). *In vivo* evidence of ER-associated process for antigen transport to the cytosol is provided by a mouse knockout for the heat shock protein 90 (HSP90) (206). HSP90 is a chaperone associated with the ERAD complex on the cytosolic side of the ER, and its knockdown in the DC-like cell line KG-1 impairs the refolding of proteins that have gained access to the cytosol (207). Soluble cytochrome *c* does not induce apoptosis in BMDCs from HSP90α-null indicating that HSP90 might participate to the cytosolic export of soluble proteins (208). Importantly, *in vivo* apoptosis of splenic cDC1s induced by cytochrome *c* injection is also impaired in HSP90α-null mice, and cross-priming of splenic CD8^+^ T cells isolated from HSP90α-null mice after immunization with OVA-coated splenocytes is reduced (206).

Finally, mice lacking the ɣ-interferon-inducible lysosomal thiol reductase (GILT) – which is present in phagosomes and lysosomes – are deficient in their ability to cross-present gB, an HSV-1 glycoprotein that contains five disulfide bonds. As the cytosolic export of ERAD substrates containing disulfide bonds requires their prior reduction and unfolding, it is feasible that GILT regulates cross-presentation by facilitating an ERAD-dependent transport of reduced antigens carrying disulfide bonds to the cytosol (209).

Of note, soluble antigens might also enter ERAD after having reached ER lumen by retrograde transport. This possibility is supported by *in vitro* studies reporting that a significant part of soluble OVA captured by moDCs actually reaches the ER lumen (210). Whether this is relevant for cell-associated antigens in cDCs remains to be established.

In summary, despite the incomplete understanding of the cellular machinery required for cytosolic transport of endocytic antigens, there is evidence indicating that cDCs have developed specific features to enhance the efficiency of cytosolic export.

## Where Does MHC Class I Loading Occur during Cross-Presentation?

Early experiments showing that cross-presentation could be inhibited by treatments like brefeldin A (149, 186), which blocks the anterograde transport of ER-synthesized proteins through the secretory pathway, led to the initial idea that MHC-I loading took place at the ER. Subsequent studies have demonstrated a fusion between the ER membrane and the phagosome at an early phagocytic step, indicating that the ER can provide a source of membrane to the phagosme for the loading of MHC-I molecules (196). Consistent with this idea, TAP, tapasin, calreticulin, Erp57, and the heavy chain of MHC class I are detected in purified early phagosomes from DCs (197). More importantly, MHC-I loaded with peptide was more abundant in phagosomes containing OVA-coupled beads, which indicates a direct loading of MHC-I molecules at the phagosome rather than a delivery of MHC-I loaded molecules to the phagosome (197). Depletion of sec22b impairs the recruitment of ER proteins like TAP2, tapasin, and calreticulin to the phagosome and cross-presentation by moDCs (199). These results point to a role of sec22 in the recruitment of essential components of the peptide-loading complex to the phagosome, the delivery of MHC-I to the phagosome does not seem to be altered by the depletion of sec22b. This is consistent with the intracellular localization of MHC-I, which at steady state is mainly found at the plasma membrane and the recycling endosome but not at the ER (211). Therefore, it is feasible to consider that part of the MHC-I loading might also occur at an endosomal compartment. Supporting this idea, the Jefferies laboratory has shown that murine DCs expressing mutated forms of the MHC-I receptor lacking a tyrosine residue of its cytoplasmic domain have an impaired MHC-I distribution and a reduction/loss in cross-presentation (212). The MHC-II chaperone might also be involved in an interaction with MHC-I and participates in the diversion of MHC-I toward endo-lysosomes (213). Whereas these results indicate that the loading of MHC-I molecules also occurs at the late endosome/lysosome, it is less clear if MHC-I loading at the endosomal compartment requires components of the cytosolic pathway or exclusively relies in the vacuolar pathway. For further discussion on MHC-I trafficking and its relevance to cross-presentation, see the accompanying review by Adiko Assi et al. in this issue.

Kurts and Burgdorf have reported that TLR engagement stimulates the recruitment of TAP and MHC-I to a mannose receptor-containing population of early endosomes in murine moDCs, specifically. Specific inhibition of TAP in early endosomes using the viral TAP luminal inhibitor US6 fused to transferrin inhibited cross-presentation but not direct presentation (which relies on ER-associated TAP) (176). By contrast, a recent study describes ERGIC-derived and sec22b-dependent TAP recruitment to bacteria-containing phagosomes as a constitutive mechanism, independent of TLR activation (214). Alternatively, TLR activation regulates cross-presentation by promoting the transport of MHC-I molecules from a recycling endosomal compartment to the phagosome. This process is mediated by the phosphorylation of SNAP23 via Myd88-dependent IKK2 activation and requires the recycling endosome GTPase Rab11 (214). Interestingly, mouse and human cDC1 cells express higher levels of Rab11, as compared to their cDC2s counterparts (71), a feature that needs further exploration to determine whether it is relevant or not for the cross-presentation of cell-associated antigens.

However, there is no doubt that cross-presentation can take place in steady state, non-activated DCs. Accordingly, DCs deficient in TLR signaling (Myd88^−/−^/TRIF^−/−^) do not show any major defects in cross-presentation (215). However, TLR3 is selectively associated with both mouse and human cDC1s (37, 47–49, 53). Accordingly, synthetic or viral agonists of the TLR3–TRIF pathway in particular, when associated with dead cells provide a strong co-activation stimulus for the cross-priming of CD8^+^ T cells specific for cell-associated antigens (89, 216). A more recent paper has shown that in Batf3^−/−^ lacking the CD103^+^ DCs CTL responses upon soluble OVA immunization and TLR3 stimulation by polyIC are impaired. Interestingly, the dependence on TLR3 expression was exclusive of cell-associated antigen, as in TLR3^−/−^ mice immunization with soluble OVA together with polyIC induced strong CTL responses depending on the dsRNA sensors MDA5/RIG-I (217).

It is rather difficult to disentangle the effects of TLR signaling on DC activation from the ones associated with antigen cross-presentation by MHC class I. Nevertheless, multiple studies suggest that TLR signaling, especially by intracellular TLRs (TLR3, 7, and 9) improve cross-presentation (218, 219).

TLR3, 7, and 9 rely on the Unc93b for their transport from ER to the endocytic pathway independently from the Golgi apparatus (220–222). Interestingly, DCs from Unc93b mutant mice were initially reported to be deficient in their ability to perform cross-presentation of cell-associated antigens (220). These findings have been challenged by a recent study that did not identify differences between wild-type and Unc93b-mutated DCs (223). Further quantitative studies might clarify this point but it is tempting to speculate that Unc93b might have a TLR-independent effect on the trafficking enabling cross-presentation, perhaps by regulating the transport of the peptide-loading complex outside of the secretory pathway.

## Concluding Remarks

The physiological relevance of the cross-presentation of cell-associated antigens is well acknowledged. Whether cross-presentation by MHC-I is a passive consequence of limited phagolysosomal digestion of dead cell bodies or something more specifically regulated remains to be understood. The identification of the molecular machineries controlling cross-presentation in cDCs should shed some light on this process.

## Conflict of Interest Statement

The authors declare that the research was conducted in the absence of any commercial or financial relationships that could be construed as a potential conflict of interest. The Review Editor Bénédicte Manoury declares that, despite having collaborated with Loredana Saveanu within the past 2 years, the review process was handled objectively.
